# Erlotinib combined with bevacizumab and chemotherapy in first line osimertinib-resistant NSCLC patient with leptomeningeal metastasis: A case report

**DOI:** 10.1097/MD.0000000000027727

**Published:** 2021-11-05

**Authors:** Musen Wang, Fuxin Zhu, Ningning Luo, Mengmeng Li, Yingxue Qi, Mingbo Wang

**Affiliations:** a Department of Pathology, Donge People's Hospital, Donge, China; b Department of Oncology, Donge People's Hospital, Donge, China.; c The Medical Department, Jiangsu Simcere Diagnostics Co., Ltd, Nanjing Simcere Medical Laboratory Science Co., Ltd, The State Key Lab of Translational Medicine and Innovative Drug Development, Jiangsu Simcere Diagnostics Co., Ltd, Nanjing, China.

**Keywords:** case report, *EGFR C797S*/sensitive mutation, first-line osimertinib resistance, leptomeningeal metastasis, NGS of cerebrospinal fluid

## Abstract

**Rationale:**

Leptomeningeal metastasis (LM) is a fatal complication of advanced non-small cell lung cancer (NSCLC) with a poor prognosis. Osimertinib is a promising option for NSCLC with LM harboring epidermal growth factor receptor *(EGFR)* mutation. However, therapeutic approaches remain a challenge for osimertinib resistant NSCLCs with LM. Although studies have reported that the first/second-generation EGFR-tyrosine kinase inhibitors were active against osimertinib-resistant NSCLC with *EGFR* C797S and sensitive mutation (SM), the resistance inevitably occurred due to the development of the *EGFR* SM/C797S/T790M triple mutations.

**Patient concerns:**

A 48-year-old woman was diagnosed with stage IV lung adenocarcinoma harboring the *EGFR* mutation in the combination of chest computed tomography, biopsy and amplification refractory mutation system-polymerase chain. One year and a half after oral administration of osimertinib, the patient progressed to extensive LM.

**Diagnoses:**

Magnetic resonance images of the brain showed extensive LM. Exfoliated tumor cells from cerebrospinal fluid (CSF) were positive detected by lumbar puncture and the cytology examination. *EGFR* mutations (exon19 E746_T751delinsI and exon20 C797S) in CSF circulating tumor DNA were detected by next-generation sequencing (NGS).

**Interventions:**

Pemetrexed (800 mg day 1), cis-platinum (40 mg day 1-3) combined with bevacizumab (400 mg day 1) every 3 weeks were administered to the patient. After 1 cycle, due to optic nerve invasion, erlotinib was applied 150 mg/d combined with previous regimen. The patient continued erlotinib monotherapy after 6 cycles.

**Outcomes:**

After LM, erlotinib combined with pemetrexed, cis-platinum and bevacizumab were administered to the patient for 4.25 months based on the CSF NGS. Then, the patient continued erlotinib monotherapy and appeared disease progression after 10 months. The overall survival is 35 months.

**Lessons:**

LM is a fatal complication of advanced NSCLC with a poor prognosis. NGS profiling of CSF circulating tumor DNA is important in NSCLC patients with LM and erotinib plus bevacizumab and chemotherapy is a promising option for patients with LM harboring *EGFR* C797S/SM.

## 1 Introduction

Leptomeningeal metastasis (LM), a fatal complication of non-small cell lung cancer (NSCLC), have increased recently due to the longer survival of NSCLC. LM is significantly more frequent in NSCLC with epidermal growth factor receptor (*EGFR*) mutations.^[1]^ Osimertinib is a promising option for NSCLC with LM harboring *EGFR* mutation regardless of T790M mutational status.^[2]^ However, therapeutic approaches remain a challenge for osimertinib resistant NSCLC patients with LM.

Liquid biopsy with cerebrospinal fluid (CSF) is a new approach, which has been used successfully in improving the diagnosis and characterization of LM.^[3]^ NSCLC with *EGFR* sensitive mutation (SM) could acquire resistance to first-line osimertinib through C797S mutation, which might respond to first- or second-generation EGFR inhibitors.^[4–6]^ Herein, we reported a patient with NSCLC acquired resistance to first-line osimertinib. LM occurred and *EGFR* C797S/exon 19 deletion (19del) mutations was detected by next-generation sequencing (NGS) of CSF circulating tumor DNA (ctDNA). The symptoms and the LM of the patient were significantly improved after erlotinib combined with bevacizumab and chemotherapy.

## 2 Case presentation

**Figure 1:**
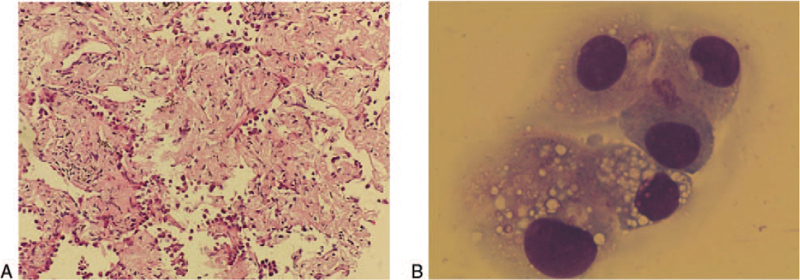
Immunohistochemistry staining of left lung puncture tumor tissue and CSF. (A) Hematoxylin and eosin staining of the puncture tumor tissue in the upper lobe of the left lung (200×). Adenocarcinoma mainly growing along the lung wall could be observed, not excluding infiltrating adenocarcinoma. (B) Cytology examination by reix-giemsa stain of cerebrospinal fluid from lumbar puncture (1000×). The exfoliated tumor cells were detected. CSF = cerebrospinal fluid.

**Figure 2:**
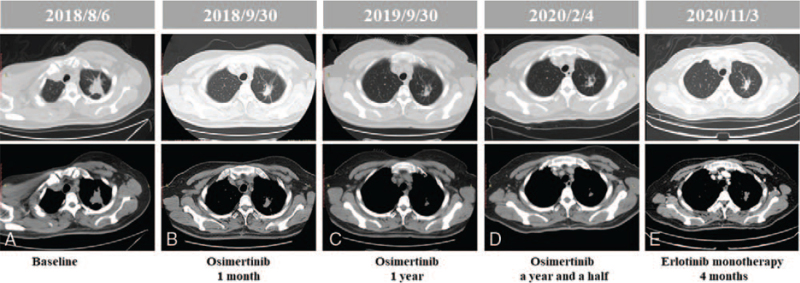
Computed tomography (CT) scan showed the changes of lung lesions during treatment. (A) Before treatment. (B) One month after osimertinib treatment, the tumor shranked. (C) One year after osimertinib treatment, the tumor shranked. (D) A year and a half after osimertinib treatment, the tumor was as before. (E) Four months after erlotinib monotherapy, the tumor had little change.

**Figure 3:**
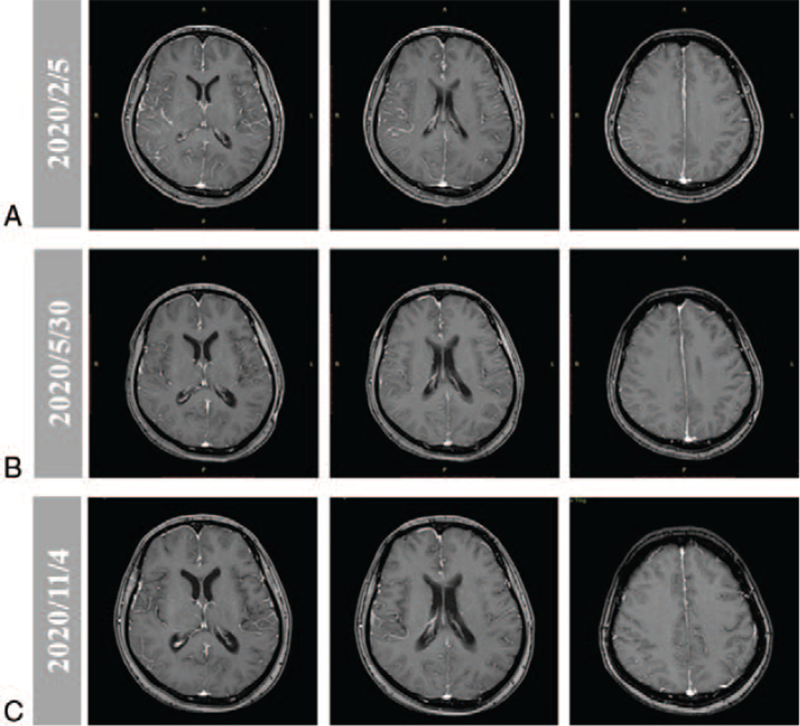
MRI image showed the changes of LM during treatment. (A) Disease progression after osimertinib a year and a half, line-like enhancement was seen in the brain. (B) Three months after erlotinib combined with bevacizumab and chemotherapy treatment, the line-like enhancement had significantly reduced. (C) Four months after erlotinib monotherapy treatment, the line-like enhancement was as before. LM = leptomeningeal metastasis, MRI = magnetic resonance images.

A 48-year-old woman presented to our hospital in August 2018 with lumbar acid distension no obvious inducement, accompanied by needle-like pain, numbness and fatigue of both lower limbs. Computed tomography (CT) scan of the chest and abdomen showed left superior lobe of lung occupied and thoracic vertebra metastasis. The spine was physically curved and the spinal cord was compressed. Under general anesthesia, T6 and T8 vertebral body was shaped; T9 pedicle screw rod was fixed systematically; and T8 fracture was reduced and spinal canal was decompressed. Biopsy of the left lung revealed stage IV adenocarcinoma via pathology (Fig. 1A) and the mutation *EGFR* 19del was detected by amplification refractory mutation system-polymerase chain. Then oral osimertinib (80 mg/d) was administered. The tumor continued to shrink, and the best efficacy evaluation was partial response (PR) during the Osimertinib treatment (Fig. 2). One year and a half later, the patient presented with a headache and vomiting, and magnetic resonance images (MRI) of the brain showed extensive LM with no obvious abnormalities in the brain parenchyma, which indicated disease progression (Fig. 3). Chest and abdomen CT scan were roughly the same as before. Lumbar puncture and the cytology examination detected exfoliated tumor cells from CSF (Fig. 1B).

**Figure 4:**
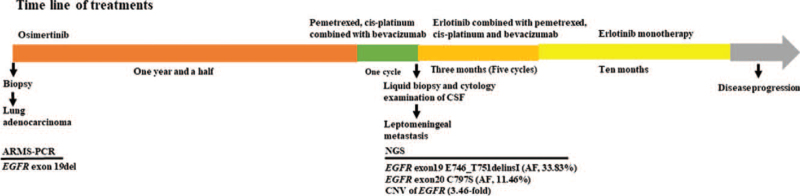
Time line of treatments of the patient undergoing and time of every treatment. The programmes of treatments were described above the consort and the pathologic type and molecular alteration of the patient were described under the consort, respectively. AF = allele frequency, ARMS-PCR = amplification refractory mutation system-polymerase chain, CNV = Copy number variations, EGFR = epidermal growth factor receptor, NGS = next-generation sequencing

Then, pemetrexed (800 mg day 1), cis-platinum (40 mg day 1-3) combined with bevacizumab (400 mg day 1) every 3 weeks were administered to the patient. After 1 cycle, the patient had a slight relief of headache, but the first-grade side reaction of blurred vision and progressive clinical aggravation occurred. Optic nerve invasion was considered after ophthalmic consultation. Evaluation of CSF ctDNA from a lumbar puncture by NGS profiling found mutations of *EGFR*, including exon19 E746_T751delinsI, exon20 C797S, and amplification (3.46-fold). Then erlotinib (150 mg/d) combined with pemetrexed (800 mg day 1), cis-platinum (40 mg day 1-3) and bevacizumab (400 mg day 1) every 3 weeks were administered to the patient. Three months later the headache symptoms of the patient were significantly relieved, and MRI of the brain showed that diffuse LM was better than before (Fig. 3). A mild rash, the first-grade side reaction, occurred after treatment and it was tolerable. Six cycles after erlotinib combined with chemotherapy and bevacizumab, the patient continued erlotinib monotherapy (150 mg/d). Four months after erlotinib monotherapy, the reexamination results of CT and MRI showed lung lesions and LM had little change, and the best efficacy evaluation was stable disease (SD) (Figs. 2 and 3). Ten months after erlotinib monotherapy (May 2021), a first-grade side reaction of decline of diet and physical strength occurred. The patient's headache significantly worsened, accompanied by vomiting. Based on these clinical symptoms, it is speculated that the LM lesion has progressed. The progression-free survival of patient administered by Osimertinib, chemotherapy and bevacizumab were 18 and 0.75 months, respectively. After LM, erlotinib combined with chemotherapy and bevacizumab were administered to the patient for 4.25 months. Then, the patient continued erlotinib monotherapy and appeared disease progression after 10 months. The overall survival is 35 months. The time line of treatments was shown in Fig. 4.

## 3 Discussion

LM is a fatal complication in patients with advanced NSCLC and the incidence is increasing, reaching 9% in *EGFR*-mutant lung cancer patients.^[7]^ The prognosis of patients with LM from NSCLC remains poor.^[8]^ The third-generation EGFR TKI osimertinib, can efficiently penetrate the blood-brain barrier and should be considered in LM with sensitizing *EGFR* mutations regardless of T790M status.^[2,9]^ However, treatment options remain a challenge for osimertinib resistant NSCLC patients with LM.

Bevacizumab and erlotinib improves PFS compared with erlotinib alone in EGFR-positive NSCLC patients.^[10]^ EGFR TKI plus antiangiogenic agent therapy, like bevacizumab or ramucirumab, might be a new option for advanced NSCLC EGFR-mutated patients.^[11]^ Erlotinib and bevacizumab is an effective treatment for NSCLC with LM and *EGFR* mutation-positive.^[12,13]^ Erlotinib penetrates into CSF easier than gefitinib and the addition of bevacizumab could enhance therapeutic effect and improve the LM.^[14,15]^ Erlotinib plus pemetrexed/cisplatin had also been reported effective in EGFR mutation patients with LM.^[16]^

*EGFR* C797S without T790M is one of the acquired resistance mechanisms in first-line osimertinib.^[17]^ Although the first- and second- generation EGFR TKIs are active for *EGFR* C797S and SM, the patient inevitably undergo development of the *EGFR* SM/C797S/T790M triple mutations.^[18]^ Now, the promising results of various combination therapy are coming out, including TKI plus chemotherapy,^[19,20]^ TKI plus bevacizumab,^[10]^ bevacizumab plus chemotherapy,^[21]^ and eventually TKI plus bevacizumab and chemotherapy.^[22]^ A neoadjuvant 4-agent combination therapy, icotinib plus carboplatin, pemetrexed and bevacizumab, for locally advanced non-squamous NSCLC patient harboring *EGFR* mutations avoiding total pneumonectomy were reported.^[23]^ Hence, we combined erlotinib, bevacizumab, pemetrexed and cis-platinum 4 drugs to avoid potential resistance.

Considering the difficulty of LM biopsy, greater efforts have been made in plasma and CSF to understand the resistance mechanisms of LM.^[24,25]^ In addition to conventional cytology examination, liquid biopsy of CSF ctDNA is the potential approach to facilitate and supplement the diagnosis of LM. Current study indicates the advantages of NGS with targeted sequencing of CSF cfDNA in *EGFR*-mutant NSCLC.^[26]^ Genotyping of CSF can indicate different response to osimertinib, reveal the genetic characteristic of osimertinib resistance in NSCLC patients with LM harboring *EGFR*-mutated.^[27]^

## 4 Conclusion

In summary, *EGFR* C797S/19del mutations were found by NGS profiling in CSF in a NSCLC patient with LM occurring after first-line osimertinib treatment. After erotinib combining with bevacizumab and chemotherapy, the symptoms and the LM were significantly improved. To our knowledge, it was the first report of first-line osimertinib-resistant NSCLC with LM harboring *EGFR* C797S/SM responding to erotinib combined with bevacizumab and chemotherapy and showed significant improved for the patient. Our case report provided a promising option for patients who were resistant to first-line osimertinib with LM.

## Acknowledgments

The authors thank Mr. Chuang Qi, Tingting Sun, Wanglong Deng, Ran Ding, and Guanghua Lu from Simceredx for the kindly assistance.

## Author contributions

All authors have made substantial contributions to the conception of the work. All authors approved the final manuscript as submitted and agree to be accountable for all aspects of the work.

**Conceptualization:** Mingbo Wang.

**Data curation:** Fuxin Zhu.

**Writing – original draft:** Musen Wang, Ningning Luo.

**Writing – review & editing:** Mengmeng Li, Yingxue Qi.
